# On the Basis of Synaptic Integration Constancy during Growth of a Neuronal Circuit

**DOI:** 10.3389/fncel.2016.00198

**Published:** 2016-08-18

**Authors:** Adriana De-La-Rosa Tovar, Prashant K. Mishra, Francisco F. De-Miguel

**Affiliations:** Instituto de Fisiología Celular-Neurociencias, Universidad Nacional Autónoma de México México, D.F., Mexico

**Keywords:** electrical coupling, synapse, leech, integration, passive conduction, development, growth

## Abstract

We studied how a neuronal circuit composed of two neuron types connected by chemical and electrical synapses maintains constant its integrative capacities as neurons grow. For this we combined electrophysiological experiments with mathematical modeling in pairs of electrically-coupled Retzius neurons from postnatal to adult leeches. The electrically-coupled dendrites of both Retzius neurons receive a common chemical input, which produces excitatory postsynaptic potentials (EPSPs) with varying amplitudes. Each EPSP spreads to the soma, but also crosses the electrical synapse to arrive at the soma of the coupled neuron. The leak of synaptic current across the electrical synapse reduces the amplitude of the EPSPs in proportion to the coupling ratio. In addition, summation of EPSPs generated in both neurons generates the baseline action potentials of these serotonergic neurons. To study how integration is adjusted as neurons grow, we first studied the characteristics of the chemical and electrical connections onto the coupled dendrites of neuron pairs with soma diameters ranging from 21 to 75 μm. Then by feeding a mathematical model with the neuronal voltage responses to pseudorandom noise currents we obtained the values of the coupling ratio, the membrane resistance of the soma (*r_m_*) and dendrites (*r*_dend_), the space constant (λ) and the characteristic dendritic length (*L* = *l*/λ). We found that the EPSPs recorded from the somata were similar regardless on the neuron size. However, the amplitude of the EPSPs and the firing frequency of the neurons were inversely proportional to the coupling ratio of the neuron pair, which also was independent from the neuronal size. This data indicated that the integrative constancy relied on the passive membrane properties. We show that the growth of Retzius neurons was compensated by increasing the membrane resistance of the dendrites and therefore the λ value. By solely increasing the dendrite resistance this circuit maintains constant its integrative capacities as its neurons grow.

## Introduction

Many neuronal circuits are fully functional at birth, long before the brain reaches its final dimensions. Brain growth after birth and during evolution imposes a major challenge to circuits, which must maintain their input output relationship as individual neurons grow. This dynamic size-scaling has several other common expressions in biology. For example, an adult Chihuahua dog is about the size of the head of a Giant Pyrenees dog. In spite of such size differences the elementary functions of their brain circuits are performed with similar accuracies. On the other hand, circuits that are well-preserved from one species to another also display wide size differences in adult specimens, for example, certain neurons performing the same function in mice or elephants have radically different dimensions. In all these cases the electrical properties of neurons and their connectivity must compensate the progressive expansion of the soma, dendrites and axons. The electrical adaptations of some individual growing neurons have been studied to a certain detail (Hochner and Spira, 1987; Kepler et al., 1990; Edwards et al., 1994a,b; Picones et al., 2003; Atkinson and Williams, 2009). Moreover, it has already been shown that the chemical synaptic strength is modulated as a circuit grows (Peng et al., 2009) and that many if not most neuronal circuits also incorporate electrical connections (Furshpan and Potter, 1959; Llinás et al., 1974; Korn and Faber, 1976; Wine and Krasne, 1982; Galarreta and Hestrin, 1999). This study was designed to find out how the input-output relationship of a neuronal circuit incorporating chemical and electrical synapses are compensated during growth.

**Figure 1:**
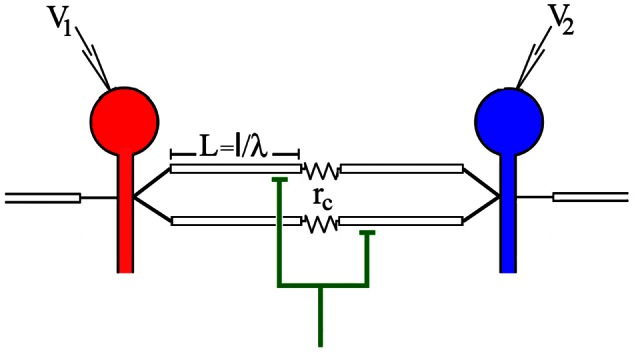
**Model of the electrically coupled Retzius cells and their common synaptic input.** The driving neuron is red and the follower neuron is blue. The voltage responses to current injection to the driving neuron were recorded from both neurons by a microelectrode inserted in each soma. The electrode for voltage recording from the driving neuron is V_1_ and the electrode for the voltage recording from the follower neuron is V_2_. The dendrites are indicated as the white cylinders with a longitude *L* = *l*/λ. Electrical synapses coupling the dendrites of both neurons are indicated as a coupling resistance (*r*_c_). The green interneuron represents the common chemical input onto the coupled dendrites of both Retzius neurons. Its activation produces the synchronous excitatory postsynaptic potentials (EPSPs) in the coupled dendrites of both neurons. The non-coupled dendrites are indicated as the open cylinders.

For this study, the central nervous system of the leech offers several advantages, since each intermediate ganglion of the central nervous system contains only about 400 neurons, most of which have been identified functionally (Blackshaw and Nicholls, 1995; Friesen and Kristan, 2007). The pair of serotonergic Retzius neurons are easily identifiable because of their largest somata in each ganglion. Each pair of Retzius neurons is coupled through an electrical non-rectifying synapse (Hagiwara and Morita, 1962; Eckert, 1963). The coupling ratio is similar among neurons in the same animal but varies from one animal to another, presumably due to intrinsic modulatory states (De-Miguel et al., 2001). This electrical synapse is established between dendrites of both neurons (Garcia-Perez et al., 2004). In addition the coupled dendrites of both neurons produce excitatory postsynaptic potentials (EPSPs) near the electrical synapse (De-Miguel et al., 2001; Garcia-Perez et al., 2004). The identity and number of neurons contributing to this input remain unidentified. However, since EPSPs of randomly-varying amplitudes are produced simultaneously in both neurons, the same input seems common to both neurons. The EPSPs produced in either neuron spread to the soma, but also cross the electrical synapse and spread to the coupled neuron where they sum with the locally-produced EPSPs to produce the action potentials that characterize the low firing frequency of these serotonergic neurons (De-Miguel et al., 2001; Garcia-Perez et al., 2004; Vazquez et al., 2009). Since the electrical synapse allows the leak of synaptic currents to the coupled neuron, the amplitude of the EPSPs is proportional to the coupling resistance value (Garcia-Perez et al., 2004; Vazquez et al., 2009). Electrical coupling of Retzius neurons with the other serotonergic neurons in the ganglion (Lent and Frazer, 1977) and also polysynaptic chemical inputs onto non-coupled dendrites of Retzius neurons have also been characterized (Velázquez-Ulloa et al., 2003), however they do not contribute to the integration form studied here (De-Miguel et al., 2001). Therefore, the simplest possible circuit is that schematized in Figure 1.

The embryonic origin of Retzius neurons has been traced to early developmental stages (Weisblat, 1981; Stuart et al., 1987) and the morphological pattern of Retzius neurons is modulated by embryonic interactions between them (Todd et al., 2010) and with their targets (Jellies et al., 1987; Loer et al., 1987). However, before birth Retzius neurons already formed their known chemical and electrical synapses (Reese and Drapeau, 1988; Todd et al., 2010; Baker and Macagno, 2014) and at the time of birth their arborization pattern is already remarkably similar to that in the adult nervous system (Jellies et al., 1987).

Using this neuronal circuit we measured the different variables contributing to synaptic integration of Retzius neurons with soma sizes ranging from 21 to 75 μm. To search for changes in the synaptic strength we studied the frequency, rise time and amplitudes of the chemical inputs acting onto the coupled dendrites of both Retzius neurons. Then we analyzed the passive properties of neuron pairs from their frequency-dependent coupling ratio obtained upon injection of pseudorandom noise current onto the driving soma, while recording the voltage from both somata. A mathematical model allowed the calculation of the passive components contributing to synaptic integration, namely the coupling resistance (*r*_c_), the space (λ) constant, the membrane resistance (*r*_m_) of the soma and dendrites (*r*_dend_) and the characteristic dendritic length (*L* = *l*/λ). The values and units of these parameters are resumed in Table 1.

**Table 1:** **Electrical parameters studied in Retzius neurons**.

Parameters		Units
*C*_m_	Specific capacitance of the membrane	μF/cm^2^
*c*_m soma_	Somatic membrane capacitance	μF
λ	Dendritic space constant	μm
*L*	Characteristic length (l/λ)	-
*l*	Dendrite length	μm
*r*_c_	Coupling resistance	MΩ
*r*_dend_	Dendritic membrane resistance	MΩ.cm
*r*_m_	Somatic membrane resistance	MΩ.cm
τ	Membrane time constant	ms

## Materials and Methods

### Preparation

Experiments were carried out in isolated ganglia obtained from leeches *Haementeria officinalis* with 15–80 mm body lengths. The somatic diameters of Retzius neurons in these ganglia ranged from 21 to 75 μm. Intersegmental ganglia were dissected out as in De-Miguel et al. (2001) and maintained in leech Ringer fluid composed of (mM): NaCl, 115; KCl, 4; CaCl_2_, 1.8; Glucose, 11; Trismaleate 10 buffered to pH 7.4 (JT Baker, México D.F., México). The somata of Retzius neurons could be unequivocally identified by their characteristic size and position in the ganglion (see De-Miguel et al., 2001). Experiments were made at room temperature (20–25°C).

### Morphology

The somatic diameters were measured from digital images of the ganglion by use of a Motic Digital Camera Plus 2.0 (China) adapted to a Nikon binocular microscope. Since most somata were elliptical, the minor and major axis of every Retzius cell were measured with Moticanplus 2.0 software. The surface area *(A)* of the soma was calculated with the relationship *A* = 4*ab*π, where “*a*” and “*b*” were the minor and major radiuses respectively. The average diameter of each spherical soma obtained as *d* = (*A*/π)^1/2^ ranged from 21 to 75 μm. The largest diameters obtained here were similar to those reported in adult animals by Garcia-Perez et al. (2004). Based on the same previous study, the length (*l*) of the coupled dendrites of each neuron was calculated by assuming isometric growth in a proportion of 0.83 times the soma diameter (Garcia-Perez et al., 2004).

### Electrophysiology

Intracellular recordings were made with borosilicate glass microelectrodes (FHC Inc, Bowdoinham, ME, USA) filled with 3 M KCl. Their tip resistances ranged from 16 to 24 MΩ. The microelectrodes were coupled to an Axoclamp pre-Amplifier (Molecular Devices: Foster City, CA, USA). Our recordings were filtered by a custom design Bessel filter with a cutoff frequency of 400 s^−1^. Data were acquired by an analog to digital board Digidata 1200 (Molecular Devices, Foster City, CA, USA) using Axoclamp 9.0 and stored in a PC for further analysis. The resting potential of neurons was between −55 and −60 mV and was held at −60 mV by injection of DC negative current when necessary. Current injection and voltage recordings from the driving neuron were made through a single electrode under discontinuous current clamp conditions with the switch operating at 0.9 KHz. Pseudorandom noise currents with 10 nA peak to peak amplitude and containing frequencies between 0.1 and 100 s^−1^ were injected through the microelectrode by a custom designed noise generator. The voltage from the follower neuron was recorded by an independent microelectrode.

Simultaneous recordings from both neurons needed to last for at least 20 min under optimal conditions, during which we repeated three times a sequence of 5 min acquisition of spontaneously-appearing EPSPs. This was followed by injection of a series of 16 square negative current pulses lasting 200 ms with gradually increasing amplitudes up to −3.0 nA to measure the input resistance of the neurons, the stationary coupling ratio and the somatic time constant. In the last part of the sequence we applied a 60 s-lasting pseudorandom noise current to determine the voltage frequency responses of each pair of neurons. The inclusion criteria for the data analysis was the value of the membrane time constant of the soma, the input resistance of the pair of neurons and the maintenance of the rise time and amplitude of spontaneous EPSPs along the whole experiment. The repetition of this whole sequence for at least three times allowed us to obtain enough EPSPs to analyze their amplitude and rise time distribution, to measure the frequency of the action potentials and the biophysical variables contributing to synaptic integration.

### Calculations of the Biophysical Parameters

The list of the parameters estimated in this study is presented in Table 1. The membrane time constant (τ) of the soma was calculated from a single exponential fitting of the passive membrane discharge of steady-state voltage responses upon the end of the square current pulses. Even though it is well-known that these discharges include information of the membrane properties of the dendritic tree, expressed as a combined series of exponentials (Rall, 1969), the large soma of Retzius neurons, which is isopotentially connected with the axon is nearly two orders of magnitude thicker than the 1 μm diameter dendrites. Therefore, the soma-axon compartment dominates the membrane charge and discharge at the recording site (Garcia-Perez et al., 2004; Vazquez et al., 2009). For our analysis we considered the specific capacitance of the membrane as *C*_m_ = 1 μF/cm^2^. The experimentally obtained τ values served then to calculate the somatic membrane resistance value (*r*_m_[MΩ.cm]) by assuming that τ = *r*_m_*c*_m_.

### Frequency-Dependence of the Coupling Ratio

The frequency-dependent coupling ratio was calculated by dividing the average power spectrum of the follower neuron by the average power spectrum of the driving neuron (Di Caprio et al., 1974; Yang and Chapman, 1983). The stationary coupling ratio was obtained from the “*y*” axis intersection at a 0.1 s^−1^ frequency.

### Estimates of Passive Properties

The values of the dendritic membrane resistance (*r*_dend_) and space constant (λ), and the coupling resistance (*r*_c_) were obtained by fitting the frequency responses of the neurons to a model of electrically-coupled Retzius neurons based on linear cable theory. The model based on the morphology of the Retzius neurons is schematized in Figure 1 (Garcia-Perez et al., 2004; Vazquez et al., 2009). In the model the soma is isopotentially connected to the axon and both are represented as a parallel circuit of membrane resistance and capacitance. The coupled dendrites are represented by cylinders with a length of 0.83 times the soma diameter (Garcia-Perez et al., 2004). One extreme of the dendrites is connected to the soma/axon compartment and the other extreme is connected to the dendrites of the coupled neuron through a resistor representing the electrical synapse. For this, the ratio of the power spectra of the pseudo-random driving and follower voltage responses to pseudorandom noise injection containing frequencies from 0.1 to 100 s^−1^ gave the frequency-dependent coupling coefficient of the neuron pair. The model was fed with the diameter and membrane resistance of the soma calculated from the steady state voltage responses to current injection. We assumed that at birth Retzius neurons already have their characteristic arborization (Jellies et al., 1987) and that their growth is isometric. Therefore, all the morphological parameters increase linearly. The dendrite length was set as 0.85 times the soma diameter and as free parameters we left the *r*_m_ and *r*_c_ values. In addition, the *r*_c_ value obtained with the model was confirmed with that obtained from the steady state measurements described above.

## Results

### Spontaneous Activity of Retzius Neurons

**Figure 2:**
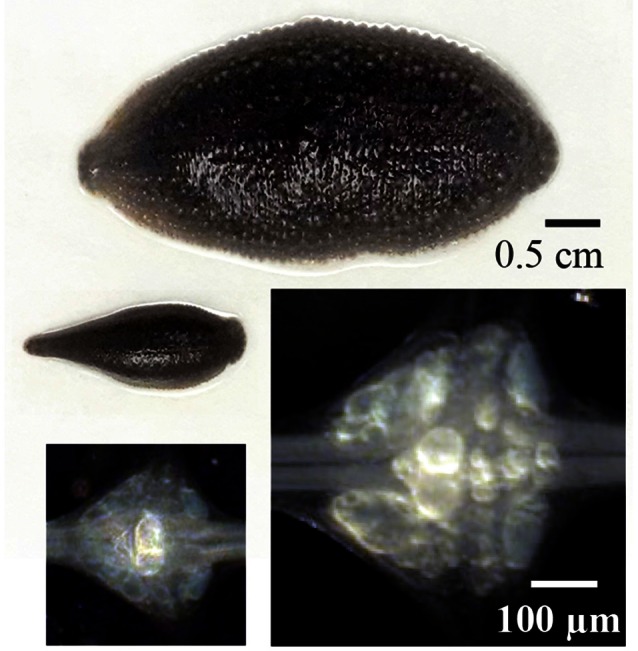
**Different sizes of leeches, their ganglia and Retzius neurons.** Above are examples large and small leeches *Haementeria officinalis* with the extreme sizes used in this study. Their central ganglia are shown below. The somata of both Retzius neurons in each ganglion are the largest and brightest by the center of each ganglion. The images of the ganglia were made by using a dark field condenser. The same scale bar applies to both leeches or to both ganglia.

**Figure 3:**
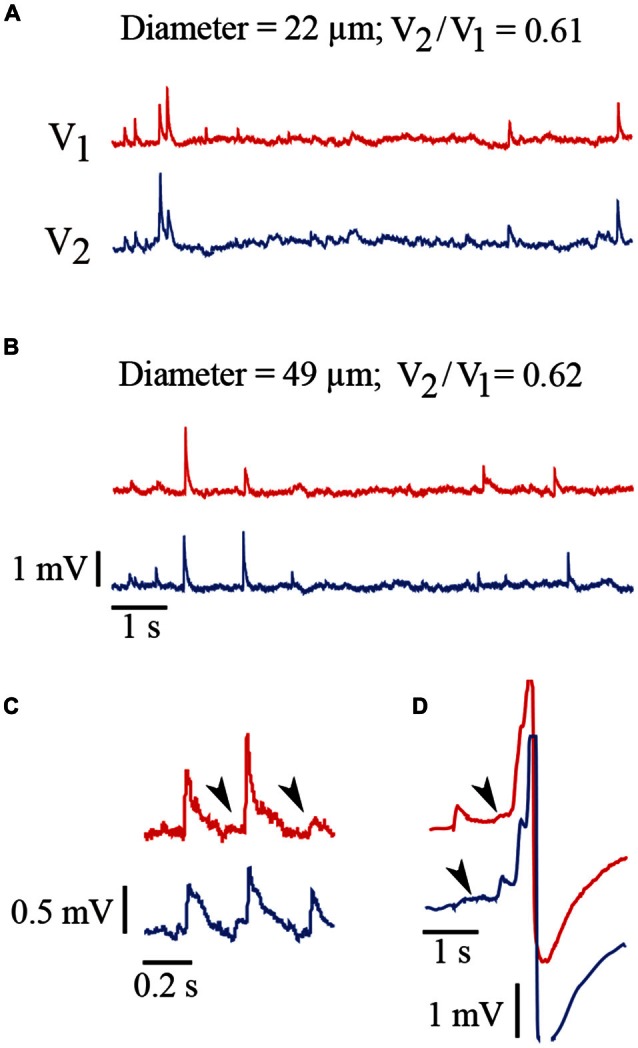
**Spontaneous electrical activity of Retzius neurons. (A)** Spontaneous synaptic activity recorded simultaneously from a pair neurons with 22 μm somatic diameters and a coupling ratio of 0.61. The record from the driving neuron (V_1_) is shown in red and the record from the follower neuron (V_2_) is shown in blue. The EPSPs produced in both neurons upon the activation of the common input appear as upwards voltage deflections. The largest spikes are produced upon summation of several EPSPs. Note the amplitude variations of the EPSPs produced simultaneously in both neurons or between subsequent EPSPs from the same neuron. **(B)** Similar voltage recordings from a pair of neurons with a 49 μm diameter and a coupling ratio of 0.62. The characteristics of EPSPs in terms of their amplitude and frequency are similar to those shown in **(A)**. The scale is the same for traces in **(A,B)**. **(C)** Amplitude variations in subsequent pairs EPSPs produced simultaneously. These amplitude differences suggest different amounts of transmitter being released from the presynaptic endings upon subsequent impulses. The arrowheads indicate the arrival of EPSPs from the coupled neuron upon a local transmission failure. Note that the propagated EPSPs are smaller and slower than those EPSPs originated in the recorded neuron. Their longer rise time and smaller amplitude are due to their spread along the dendrites from their site of origin in the coupled neuron and across the electrical synapse (De-Miguel et al., 2001). **(D)** EPSPs produced in both neurons sum and contribute to produce action potentials. Again the arrows indicate EPSPs arriving from the coupled neuron.

Examples of intact leeches with the smaller and larger sizes used in this study are shown in Figure 2. Their isolated ganglia are also shown displaying the somata of the Retzius neurons. The overall electrical activity recorded from nine pairs of these Retzius neurons with average soma diameters between 21 and 75 μm was similar to that already described in adult neurons (Hagiwara and Morita, 1962), consisting of a resting potential between −55 and −60 mV, on top of which pairs of EPSPs appeared simultaneously in both somata with amplitudes that varied randomly between neurons and from one pair to the next (Figures 3A–C), thus suggesting presynaptic stepwise variations in transmitter release (De-Miguel et al., 2001). Summation of EPSPs produced in the dendrites of both Retzius neurons produced the action potentials that characterized the basal activity of Retzius neurons (Figure 3D).

**Figure 4:**
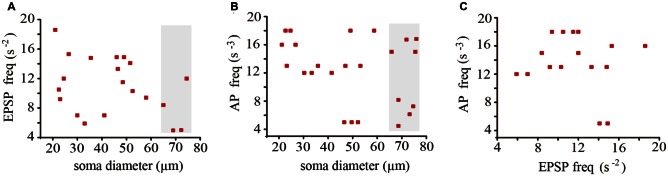
**Average frequencies of EPSPs and action potentials recorded from 18 neurons forming nine pairs with different soma sizes.** The frequency of EPSPs **(A)** and action potentials **(B)** did not correlate with the neuron diameter. Data in the gray areas are from adult neurons. **(C)** The action potential and the EPSP frequencies were also uncorrelated.

In neurons held at −60 mV the EPSPs occurred at average frequencies ranging from one neuron pair to another between 0.5 and 1.7 s^−1^, again without any correlation with the soma diameter (Figure 4A). The action potentials that complement the baseline electrical activity of these neurons were produced with average frequencies of 0.004–0.0017 s^−1^ upon summation of the EPSPs originated in both coupled neurons (Figure 4B). However, the action potential frequency did not correlate with the EPSPs frequency (Figure 4C), presumably because summation was multivariable and included the proportion of local and propagated EPSPs in each pair of neurons, the individual EPSPs arriving from the coupled dendrites, the stochastic presynaptic amplitude fluctuations, and as will be seen below, the modulation of the EPSP amplitude upon the leak of synaptic currents across the coupling resistance.

**Figure 5:**
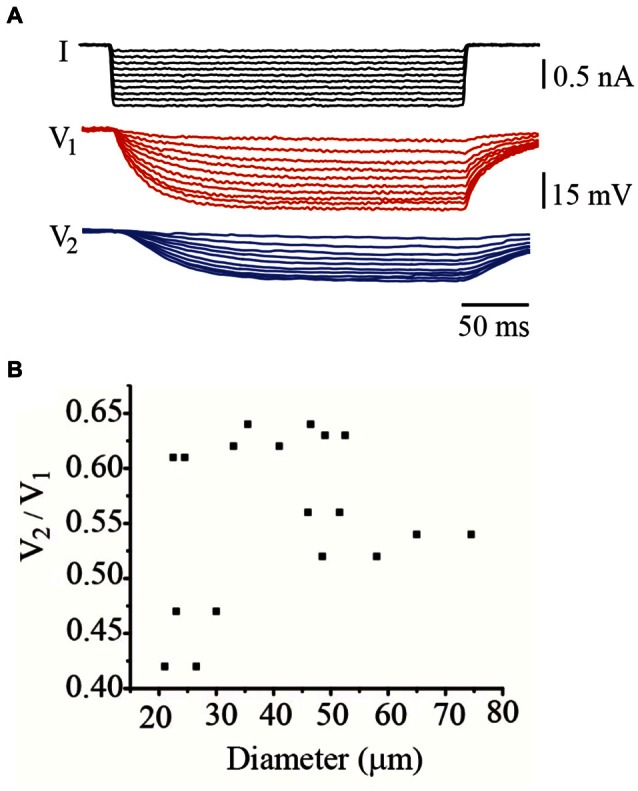
**The coupling ratio varied independently on the neuron diameter. (A)** Steady state voltage responses produced by hyperpolarizing current steps injected in the driving neuron. Voltage was recorded simultaneously from the driving (V_1_; red traces) and from the follower (V_2_; blue traces) neurons. **(B)** The coupling ratio was independent from the soma diameter. Note that the range of coupling ratios is smaller than that in adult neurons.

A third parameter contributing to integration in these neurons is their electrical coupling. In adult neurons the coupling ratio calculated from steady state voltage responses upon square current pulses (Figure 5A) ranges between 0.2 and 0.7 (Garcia-Perez et al., 2004). In these experiments, the coupling ratio had a smaller range presumably because the unpredictability of its variations from leech to leech. However, the coupling ratio of individual neuron pairs failed to correlate with their soma diameter (Figure 5B). For example, neurons from the two smaller pairs studied, having 21 and 26 μm soma diameters, had coupling ratios of 0.42 and 0.64, respectively, whereas neurons with a 36 μm diameter also had a high 0.64 coupling ratio. By contrast, the largest neuron studied here had a 75 μm diameter and an intermediate 0.53 coupling ratio.

**Figure 6:**
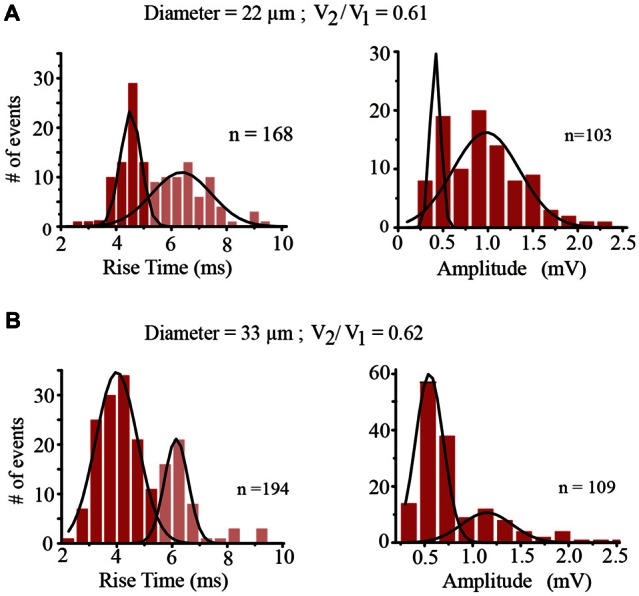
**Characteristics of EPSPs. (A)** Rise time (left) and amplitude (right) distributions of EPSPs recorded from the same neuron with a 22 μm diameter and a coupling ratio of 0.61 shown in **(A)**. The rise time of EPSPs indicates their spreading distance from their origin to the soma. EPSPs with a faster rise time (red) were produced in the dendrites of the driving neuron whereas the EPSPs with slower rise times (pale red) were produced in the coupled dendrites of the follower neuron and arrived at the soma of the driving neuron after spreading across the electrical synapse (De-Miguel et al., 2001; Garcia-Perez et al., 2004). The “*n*” values indicate the number of events in the distribution plots. The amplitude distributions were produced using only the fast-rising EPSPs generated in the dendrites of the driving neuron. The distribution displayed two amplitude peaks, the second of which doubled the amplitude of the first. **(B)** Similar rise time and amplitude distributions were obtained from a neuron pair with a 33 μm diameter and a similar coupling ratio of 0.62. In spite of the size differences of the neuron pairs. EPSPs arriving at the somata of neuron pairs with similar coupling ratios had similar amplitudes.

**Figure 7:**
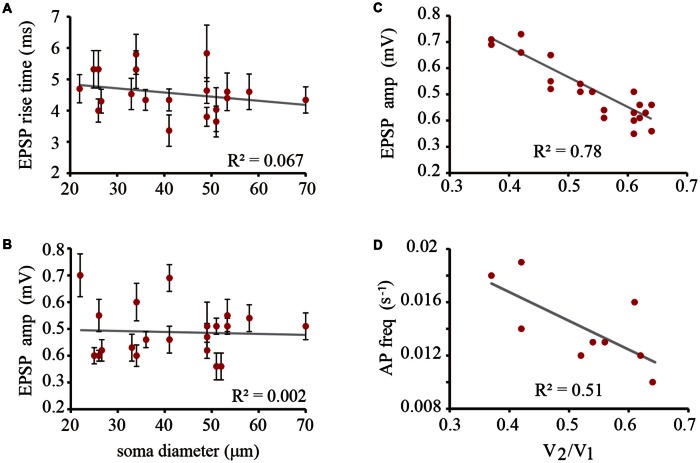
**The EPSP amplitude and the action potential frequency depended on the coupling ratio. (A)** The rise time of EPSPs did not correlate with the soma diameter. **(B)** The amplitude of the unitary peak of EPSP amplitude did not correlate with the soma diameter. **(C)** The amplitude of local EPSPs is inversely proportional to the coupling ratio of the neuron pair. This effect is due to the current leak of the coupled neuron across the electrical synapse. **(D)** The action potential frequency is inversely dependent on the coupling ratio of the neuron pair presumably because of the summation of ESPS. The gray lines are the predicted linear fittings to the data. The correlation coefficient is indicated in each plot. The low correlation coefficient of the action potential frequency may be due to the multivariable summation that is affected by the amplitude fluctuations between individual EPSPs, summation with EPSPs from the coupled neuron and the frequency variations from neuron to neuron. All these variables are independent on the neuron size.

The amplitude and rise time of EPSPs also provide information about integration in terms of their presynaptic contents, their origin in the dendrites and their spread along the dendrite to the soma and their spread to the coupled neuron across the electrical synapse (De-Miguel et al., 2001; Garcia-Perez et al., 2004). The rise time distribution of EPSPs in every neuron had two peaks, with the value of each peak indicating the EPSPs origin in the coupled dendrites (Figure 6). Those EPSPs with fast rise times (59–81%, *n* = 9 neurons) ranging from 3.63 ± 0.5 to 5.83 ± 0.9 ms are produced in the coupled dendrites of the driver neuron, whereas the EPSPs (19–41%) with slower rise times between 7.33 ± 0.6 and 9.13 ± 1.3 ms (*n* = 9) and smaller amplitudes (not shown, De-Miguel et al., 2001) are originated in the dendrites of the coupled neuron, from which they arrived after crossing the electrical synapse (De-Miguel et al., 2001; Garcia-Perez et al., 2004). We have shown that the slower EPSPs are detected upon local failures in transmitter release onto the driving neuron, since otherwise they appear as a hump in the decay phase of the local EPSPs upon summation in the primary axon (Vazquez et al., 2009). The ranges of EPSP rise time values and their proportions were similar to those already described in adult neurons (Garcia-Perez et al., 2004), and had no correlation with the soma diameter (0.067; Figure 7A) thus suggesting that in spite of the dendrite elongation, the EPSP electrotonic spread remains constant as neurons grow.

The amplitude distribution of the local EPSPs obtained from Gaussian fittings to the fast-rising EPSP population recorded from the nine pairs plus three other neurons (Figure 6) had one peak formed by the smaller events (*n* = 21 neurons); a second peak (*n* = 19 neurons) had events whose amplitude nearly doubled that of the first peak, and in records from two neurons a third small peak contained even largest EPSPs. These distributions along with the transmission failures onto each neuron suggested a quantal nature in the EPSP amplitudes, with the smaller amplitude peaks being produced by the unitary events.

### Modulation of the EPSP Amplitude by Electrical Coupling

The unitary EPSP amplitude varied from neuron to neuron between 0.36 ± 0.05 and 0.7 ± 0.8 mV. However these variations were not correlated with the soma diameter (*R*^2^ = 0.03; Figure 7B). Instead of that and consistent with our previous prediction (Garcia-Perez et al., 2004) the EPSP amplitude was inversely proportional to the coupling ratio of the neuron pair (Figure 7C) with a 0.78 correlation coefficient. The explanation for this effect is that electrical coupling modulates the amplitude of the EPSPs by allowing a synaptic current leak in proportion to the coupling resistance value. Indeed, the EPSP amplitude was similar in neurons with similar coupling ratio. For example, in a small neuron with a 22 μm soma diameter and a 0.61 coupling ratio, the EPSP distribution had a major 0.45 ± 0.05 mV amplitude peak, whereas a larger neuron in another pair with a 33 μm soma diameter and a similar 0.62 coupling ratio, produced unitary EPSPs with a 0.43 ± 0.5 mV amplitude. The variability increases of the data at high coupling ratios shown in Figure 7 could be due to contamination by the 0.18 mV root-mean-square (RMS) noise in the recordings and to a higher susceptibility of smaller EPSPs to input resistance differences and to the multivariable influences on their spread. Consistently with the behavior of EPSPs, the frequency of the action potentials decreased as the coupling ratio increased. The low correlation coefficient of 0.5 of the plot shown in Figure 7B can be due to the multiple variables influencing EPSP summation. These data confirm that at similar coupling coefficients the membrane properties of the neurons compensate their size to produce constant integrative properties.

### Estimates of Passive Electrical Properties

**Figure 8:**
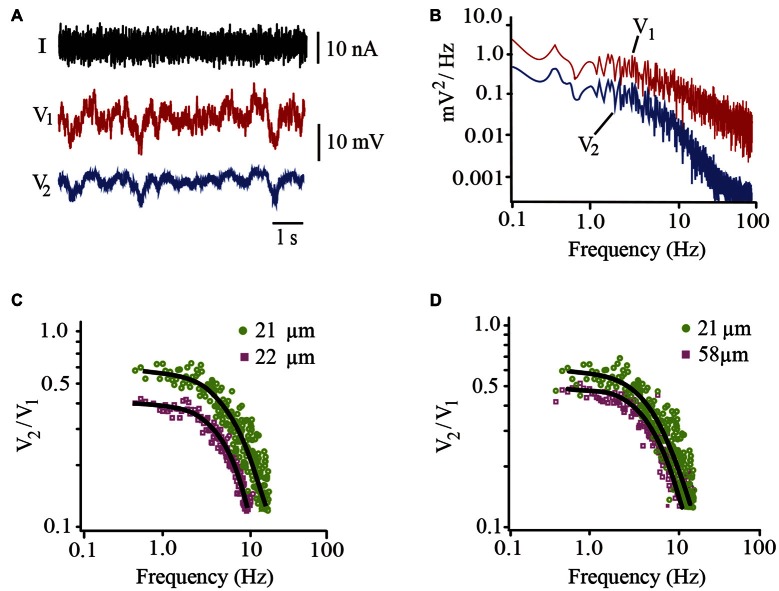
**Estimates of biophysical parameters contributing to integration. (A)** Non-stationary voltage responses recorded from the driving (V_1_; red) and follower (V_2_; blue) neurons upon injection of pseudorandom noise current (*I*) into the driving neuron. **(B)** Power spectra obtained from the Fourier analysis of the driving and follower neuron voltages upon injection of noise currents. **(C)** Frequency-dependent coupling ratio obtained by dividing the power spectra of the follower neuron by that of the driver neuron. Data are from two neuron pairs with similar 21 and 22 μm diameters but different steady-state coupling ratios of 0.42 (purple) and 0.6 (green), respectively. The continuous black curves are the best model fits to the data. Note the differences in the predicted intersections with the coupling ratio axis. **(D)** Plots obtained from neuron pairs displaying 21 and 58 μm diameters and 0.6 and 0.5 coupling ratios.

In addition to the somatic membrane resistance and capacitance of the soma estimated from the voltage changes upon hyperpolarizing square current pulses (Figure 5), the frequency-dependent coupling ratio was calculated by injecting pseudo-random noise current into the driving neuron (Figure 8A). The power spectrum of the follower neuron was then divided by the average power spectrum of the driving neuron (Figure 8B) to obtain the frequency-dependent coupling ratio (Figures 8C,D). Model fitting to the data provided a calculation of the λ value of the dendrites and confirmed the somatic membrane time constant. In addition, the coupling ratios ranging from 0.41 to 0.64 obtained from steady-state responses correlated linearly (*R* = 0.96) with those obtained from noise analysis extrapolating the model fitting to a 0.1 s^−1^ frequency.

### The Membrane Resistance of the Dendrites Compensated for their Growth

**Figure 9:**
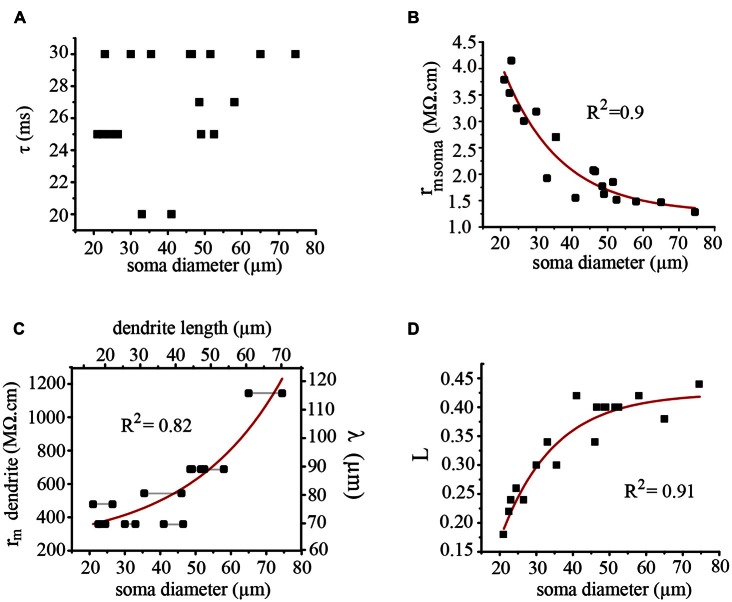
**Electrical parameters of the soma and dendrites contributing to synaptic integration. (A)** The somatic time constant of neurons with different soma diameters was restricted to 20–30 ms. **(B)** The membrane resistance of the soma decreased exponentially as the soma diameter was increased. **(C)** The membrane resistance of the dendrites (*r*_m_) and their λ values increased as the soma diameter and the dendrite length increased. The dendrite length was calculated considering an isometric growth of the dendrites and the soma. The horizontal black lines indicate neurons forming a pair. **(D)** The *L* value (*l*/λ) increased exponentially as the soma diameter increased. The correlation (*R*^2^) values of the curve fittings are indicated for each case.

The time constant values measured from the exponential voltage decay had a scattered distribution between 20 and 30 ms for the whole range of somatic diameters (Figure 9). This range was within the 18–40 ms range obtained in our previous experiments in isolated adult somata (Garcia-Perez et al., 2004), thus suggesting that the correction factor for growth was in the coupled dendrites. It is worth to remark here that the large soma-axon dominates the voltage responses of these neurons upon current injection, thus making the contribution of the dendrites negligible in soma charging experiments. Therefore these voltage responses are expressed as a single exponential change, as compared with the dual exponentials that characterize the somatic voltage changes in the presence of small somata and large dendritic trees (Rall, 1959). As expected, the membrane capacitance (*c*_m_) values of the soma-axon compartment increased linearly (*R* = 0.99) with the soma diameter, from 6.6 pF when the diameter was 21 μm to 23.4 pF when the soma diameter was 75 μm. By contrast, the soma membrane resistance (*r*_m_) decreased exponentially (*R*^2^ = 0.9) from 3.4 MΩ.cm when the diameter was 21 μm to 1.4 MΩ.cm when the soma diameter was 74 μm (Figure 9B). Therefore the variability of the somatic time constant values was produced by the combination of the linear capacitance increase and the exponential resistance decrease as the soma increased.

The parameter compensating for the neuron growth was the membrane resistance of the dendrites, which displayed an exponential increase (*R*^2^ = 0.82) as the somatic diameter was larger (Figure 9C). By assuming a realistic constant value of 85 KΩ/cm for the internal resistance of the dendrites (Garcia-Perez et al., 2004) the exponential increase in the *r*_dend_ value also produced an increase in the dendritic λ value (Figure 9C, right axis). Therefore, the effective length *L* = *l*/λ of the dendrites increased exponentially (*R*^2^ = 0.91) as the soma diameter increased (Figure 9D) to reach a plateau when the soma approached a 50 μm diameter.

## Discussion

The microcircuit established by electrically-coupled Retzius neurons and their chemical input maintains its integrative properties as the neuronal soma of neurons triples its diameter during growth. By fitting a model to our experimental data, we measured different variables with relative independence from each other in the same neuron pair. The coupling ratio of the pairs of Retzius neurons varies within a broad range of values from small and adult neurons and these variations are uncorrelated to the neuronal size, as it happens in adult animal. The frequency and the characteristics of the EPSPs that produce the basal firing frequency of Retzius neurons are already determined in the earliest postnatal stages tested in our study and remained unchanged as neurons grow. This requires that the presynaptic inputs are already mature as neurons grow. Postsynaptically, the increase in the membrane resistance of the dendrites was sufficient to compensate for the morphological growth of the circuit, thus permitting the production of EPSPs with similar characteristics in the whole range of neuronal sizes. This effect produces constancy in the basal integrative capacities as the circuit grows. The circuit has in addition different modulatory influences from early stages, including the presynaptic variations on the frequency of EPSP and their amplitude variations, and the postsynaptic changes in the coupling ratio.

The increase in the dendritic membrane resistance during neuron grow is counterintuitive since an increase in the membrane area conveys a decrease in the specific membrane resistance. Therefore, this resistance increase implies a local reduction in the number of effective channels per unit of dendrite membrane. There are several possible ways to achieve such effect. One is through a gradual reduction in the open state probability or the conductance of the channels determining the membrane resistance as neurons grow. Such type of modulation exists in two-pore domain potassium channels, which are major contributors to the resting potential and the membrane resistance of neurons and muscle cells (Goldstein et al., 2001). Another possibility is a selective reduction in the incorporation rate of resting-active channels to the dendrites. Dendrites synthesize proteins that are incorporated in their membrane (Kiss, 1977; Slomnicki et al., 2016). In any case, this resistance reduction should be local, since the soma and axon membranes conserve their time constant as neurons grow, this being consistent with a resistance reduction proportional to the capacitance increase.

### Integrative Properties of Growing Neurons

The persistency of the axo-somatic time constant as neurons grow imposes a similar decay to EPSPs arriving at the primary axon-soma compartment independently on the neuron size. Since the frequency range at which EPSPs appear similar as neurons grow, the temporal summation that determines the basal firing frequency also remains constant provided amplitudes of EPSPs are similar. Therefore, the variable determining the firing frequency at all neuronal sizes is the coupling ratio of the neurons. A second factor contributing to synaptic integration is the small dendro-somatic conductance coefficient, which reduces significantly the amplitude of EPSPs upon arrival at the axon (Vazquez et al., 2009). Although this coefficient changes the amplitude of all the EPSPs but not their shape, the remodeling of the EPSPs entering the soma/axon compartment determines how they sum (Vazquez et al., 2009). Our data indicates a constant dendro-somatic coefficient since neurons of different sizes but similar coupling ratios had similar EPSP amplitudes and firing frequencies. It was surprising that the electrical coupling values of small neurons spans over a similar wide range as adult neurons. This result indicates that from early postnatal life electrical coupling has already its modulatory capacity to regulate the basal firing frequency of the neurons through the regulation of the EPSP amplitude.

### Functional Implications for Serotonergic Neurons

Serotonergic neurons of vertebrates and invertebrates are multimodal in the sense that they release transmitter from synapses or from extrasynaptic sites, depending on their electrical activity pattern (De-Miguel et al., 2015). In general, serotonergic neurons display spontaneous firing at low frequencies (Mosko and Jacobs, 1974; Wang and Aghajanian, 1977; Cunningham and Lakoski, 1988; Mason, 1997; Veasey et al., 1997), which do not evoke extrasynaptic transmission. Even the smallest Retzius neurons studied here had established the pattern of EPSPs generation that characterizes adult neurons. Summation of EPSPs produces the low firing frequency of Retzius neurons (Garcia-Perez et al., 2004). The early establishment of this pattern implies that from early life this circuit has acquired the basal firing frequency that will continue during adulthood, in contrast to the gradual increase in the number of synaptic inputs in cricket or rat neurons as they grow (Chiba et al., 1992; Liu et al., 1996). At their basal firing frequency, Retzius neurons may release serotonin from synaptic endings (Henderson, 1983; Liu and Nicholls, 1989). However, serotonergic neurons also release serotonin extrasynaptically from their soma, dendrites and varicosities when the firing frequency increases (Trueta et al., 2003; Leon-Pinzon et al., 2014) and by doing this may modulate whole circuits (De-Miguel et al., 2015). This extrasynaptic exocytosis requires another set of excitatory connections from mechanosensory neurons (Szczupak and Kristan, 1995; Velázquez-Ulloa et al., 2003). Firing of pressure sensory neurons upon skin stimulation produces a proportional firing increase in adult Retzius neurons (Velázquez-Ulloa et al., 2003). The massive serotonin release from the soma increases the levels of circulating serotonin in the ganglion (Willard, 1981) and evokes fictive swimming (Nusbaum and Kristan, 1986) or crawling (De-Miguel et al., 2015). A whole scheme to understand serotonergic function will require a comprehensive analysis of the development of this alternative pathway. Another question concerns the modulation of electrical coupling. In serotonergic neurons this modulation may be produced by serotonin release (Colombaioni and Brunelli, 1988; Rorig and Suttor, 1996; Szabo et al., 2010) acting on the gap junction channels (Phelan et al., 1998; Kandarian et al., 2012). However Retzius neurons from small animals are insensitive to serotonergic modulation (Groome et al., 1995), thus suggesting on one hand that the extrasynaptic communication may not yet be functional and on the other that the modulation of gap junctions is produced by a different and yet unknown mechanism.

## Author Contributions

FFD-M conceived the experiments. AD-L-RT, PKM and FFD-M designed, performed the experiments and analyzed the data. FFD-M contributed reagents/materials/analysis tools. FFD-M wrote the article.

## Conflict of Interest Statement

The authors declare that the research was conducted in the absence of any commercial or financial relationships that could be construed as a potential conflict of interest.
