# Corrigendum: On the Basis of Synaptic Integration Constancy during Growth of a Neuronal Circuit

**DOI:** 10.3389/fncel.2017.00399

**Published:** 2017-12-12

**Authors:** Adriana De-La-Rosa Tovar, Prashant K. Mishra, Francisco F. De-Miguel

**Affiliations:** Instituto de Fisiología Celular-Neurociencias, Universidad Nacional Autónoma de México, Ciudad de Mexico, Mexico

**Keywords:** electrical coupling, synapse, leech, integration, passive conduction, development, growth

Correction to the acknowledgments:

Adriana De-La-Rosa Tovar and Prashant Kumar Mishra were Doctoral Students of the Doctorado en Ciencias Biomédicas at the Universidad Nacional Autónoma de México.

The original article has been updated.

## Conflict of interest statement

The authors declare that the research was conducted in the absence of any commercial or financial relationships that could be construed as a potential conflict of interest.

